# 12q14.3 microdeletion involving HMGA2 gene cause a Silver-Russell syndrome-like phenotype: a case report and review of the literature

**DOI:** 10.1186/s13052-020-00866-9

**Published:** 2020-07-28

**Authors:** Francesca Mercadante, Martina Busè, Emanuela Salzano, Tiziana Fragapane, Daniela Palazzo, Michela Malacarne, Maria Piccione

**Affiliations:** 1 U.O.S.D. Medical Genetics, AOOR Villa Sofia-Cervello, Palermo, Italy; 2 U.O.C. Laboratory of Human Genetics, IRCCS G. Gaslini, Genoa, Italy; 3 Department of Health Promotion, Mother and Child Care, Internal Medicine and Medical Specialties, University of Palermo, Palermo, Italy

**Keywords:** Silver-Russell syndrome, Failure to thrive, *HMGA2* gene, Netchine-Harbison clinical scoring system, Case report

## Abstract

**Background:**

Silver-Russell Syndrome (SRS) is a genetic disorder characterized by intrauterine and postnatal growth restriction and normal head circumference with consequent relative macrocephaly. Addictional findings are protruding forehead in early life, body asymmetry (of upper and lower limbs) and substantial feeding difficulties. Although several genetic mechanisms that cause the syndrome are known, more than 40% of patients with a SRS-like phenotype remain without an etiological diagnosis. In the last few years, different clinical reports have suggested that mutations or deletions of the *HMGA2* gene can be responsible for a SRS-like phenotype in patients with negative results of the common diagnostic tests for this syndrome.

**Case presentation:**

We present a 3-year-old male patient with clinical diagnosis of Silver-Russell Syndrome (SRS) associated with a de novo heterozygous deletion of the long arm of the chromosome 12 (12q14.3) encompassing the *HMGA2* gene.

**Conclusions:**

Our report confirms the etiological role of *HMGA2* as a disease gene in the development of a SRS-like phenotype.

## Background

Silver-Russell Syndrome (SRS) is a distinct syndromic growth disorder in which prenatal and postnatal growth failure are associated with other characteristic features, including relative macrocephaly at birth, protruding forehead in early life, body asymmetry and substantial feeding difficulties. Almost all children with SRS are born SGA. Postnatal catch-up growth is not seen in the majority of children with SRS [1].

Multiple clinical scoring systems have been proposed for Silver-Russell syndrome (SRS). In their *Prospective Study Validating a Clinical Scoring System and Demonstrating Phenotypical-Genotypical Correlations in SRS*, Azzi et al. proposed a new scoring system (Netchine-Harbison clinical scoring system), processing a flow-chart for investigation and diagnosis of SRS [2].

SRS can be caused by different genetic mechanisms. The most common abnormalities are related to the epimutation of either the 11p15.5 region or the chromosome 7. In particular, the H19/IGF2:IG-DMR hypomethylation occurs in almost 40% of cases, followed by the maternal uniparental disomy of chromosome 7 (4–10% of cases), the maternal uniparental disomy of 11p15.5 region (less than 1% of cases), and the multilocus hypomethylation with or without ICR2 hypomethylation (less than 1% of cases). Chromosomal rearrangements, such as the duplication of maternal 11p15.5 region and other cryptic chromosomal aberrations, have been reported in less than 1% of cases respectively. However, all these genetic aberrations can be detected in less than 60% of SRS cases, and thus the clinical diagnosis remains without a genetic etiology [1–5].

In the last decade, several studies have suggested the role of *HMGA2* as candidate gene in those patients with SRS phenotype and negative result of classical genetic tests for SRS. *HMGA2* gene encodes for the HMGA2 protein, member of the “high-mobility group AT-hook” (HMGA) family. These proteins act as architectural transcription factors that regulate the trascriptional activity of several genes. The expression of HMGA proteins is high in early developmental stages in embryos and mesenchymal stem cells, whilst it is almost absent or very low in adult tissues. Additionally, several studies have showed their role in adipocytes differentiation and their overespression in some tumoral tissues [6–8].

In 2007, Menten et al. [9] reported SRS patients with the microdeletion of the 12q14.3 region encompassing different genes including *HMGA2* and *LEMD3* (an OMIM Disease Causing gene associated with Buschke-Ollendorff syndrome and Osteopoikilosis with or without melorheostosis). To our knowledge, other three heterozygous pathogenic variants and two exon deletions involving the *HMGA2* gene have been described in patients with clinical diagnosis of SRS [8, 10–12].

We report the clinical and genetic characteristics of a patient with SRS phenotype and a de novo 425 Kb microdeletion of 12q14.3 region encompassing the *HMGA2* gene but not the *LEMD3* gene.

## Case presentation

Our patient is a 3-year-old boy, third child of Caucasian, non-consanguineous and healthy parents. He was born at 39 weeks of gestation via natural delivery, after an unremarkable pregnancy. At birth he was small for gestational age: weight of 2350 g (<3rd centile), length 47 cm (2nd centile), head circumference (HC) 31 cm (<3rd centile). His Apgar scores were 9 at 1 min and 10 at 5 min. His past medical history was significant for a persistent failure to thrive during his first year of life. Several investigations, including a work-up for metabolic and gastrointestinal disorders, multiple urine tests, cranial ultrasound, echocardiogram and abdominal ultrasound, were normal. Endocrinological assessment showed a normal growth hormone (GH) function, but a moderate hypothyroidism, still treated with levothyroxine. At 8 months of age audiological evaluation was normal, while ophtalmologic exam revealed mild pseudostrabismus.

He was referred to our attention at the age of 10 months. His anthropometric parameters were: weight 5900 g (< − 2 SDS), length 64 cm (< − 2 SDS), head circumference (HC) 43.8 cm (9th – 25th centile). In addition, physical examination showed: triangular facies, closed anterior fontanelle, wide forehead, frontal bossing, deeply set eyes, epicanthus inversus, downslanted palpebral fissures, blue sclerae, narrow chin with slight vertical crease, ears posteriorly rotated with prominent anterior crus of antihelix and underdeveloped tragus and antitragus, syndactyly of 2th/3th finger of the foots, clinodactyly of 5th finger of the hands, low muscle mass, asymmetric lower limbs (left almost 1 cm shorter than right). A neuropsychiatric consult was requested, and then a moderate speech delay was detected. The brain MRI was normal.

**Fig. 1:**
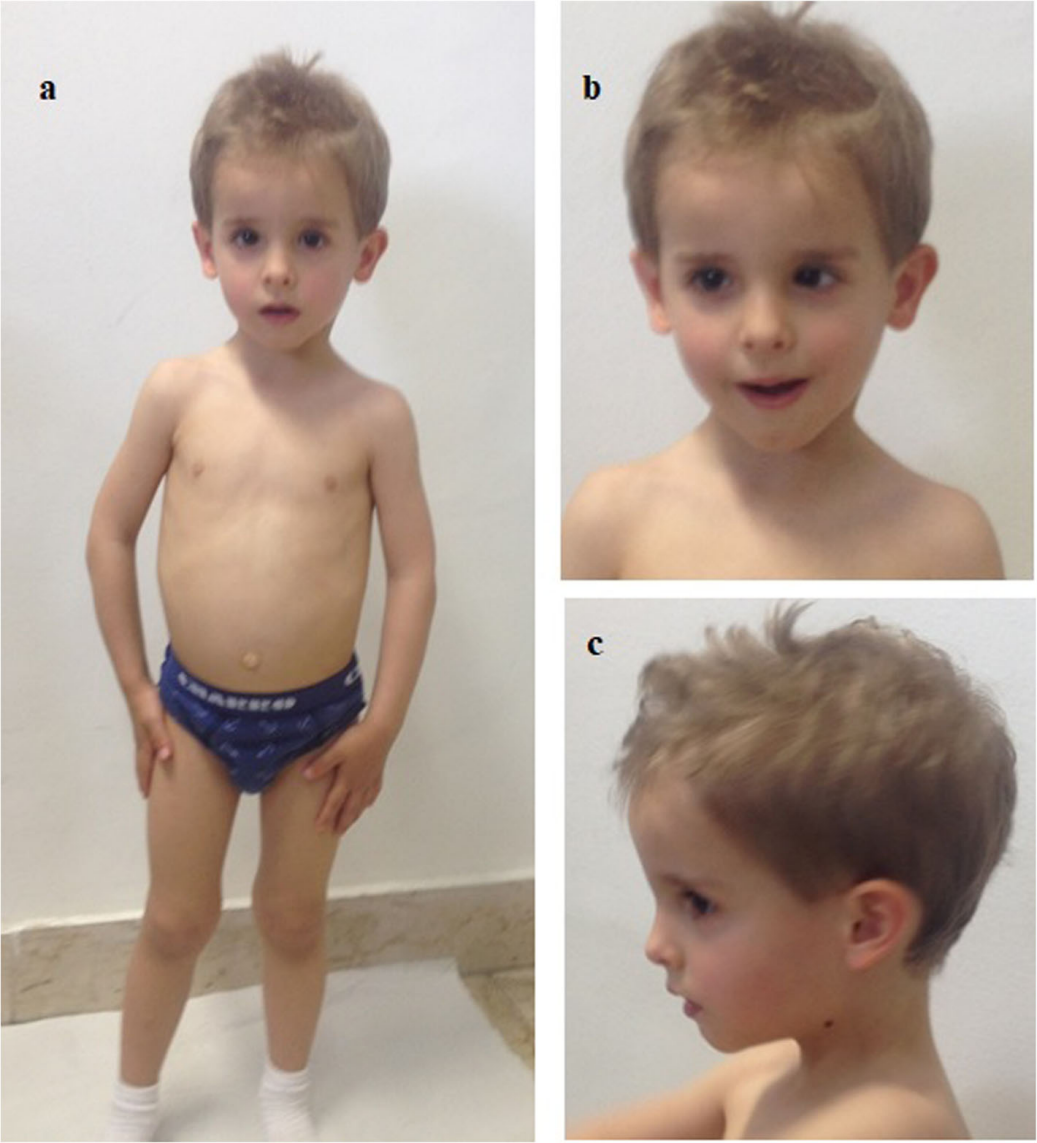
**a**, **b**, **c** Our patient at 3 years and 4 months of age. Note triangular facies, wide forehead, frontal bossing, deeply set eyes, epicanthus inversus, downslanted palpebral fissures, narrow chin with slight vertical crease, ears posteriorly rotated with prominent anterior crus of anthelix and underdeveloped tragus and antitragus

At 2 years, the clinical picture appeared unchanged. His weight was 7500 g (<3rd centile), his lenght was 81 cm at the left side and 80 cm at the right (<3rd centile), his HC was 47,3 cm (10rd centile) and his BMI was 11,7 (<3rd centile). During the last check-up at our Center, at the age of 3 years, no significant changes were revealed regarding the clinical presentation and growth parameters. His weight was 9450 g (<3rd centile), his lenght was 88 cm at the left side and 89 cm at the right (3rd centile), and his HC was 48,2 cm (3rd centile) (Fig. 1).

Applying the Netchine-Harbison CSS (NHCSS) we got a score of 5/6 (the proband met all the criteria for likely SRS except for relative macrocephaly at birth) [2].

### Genetic tests

**Fig. 2:**
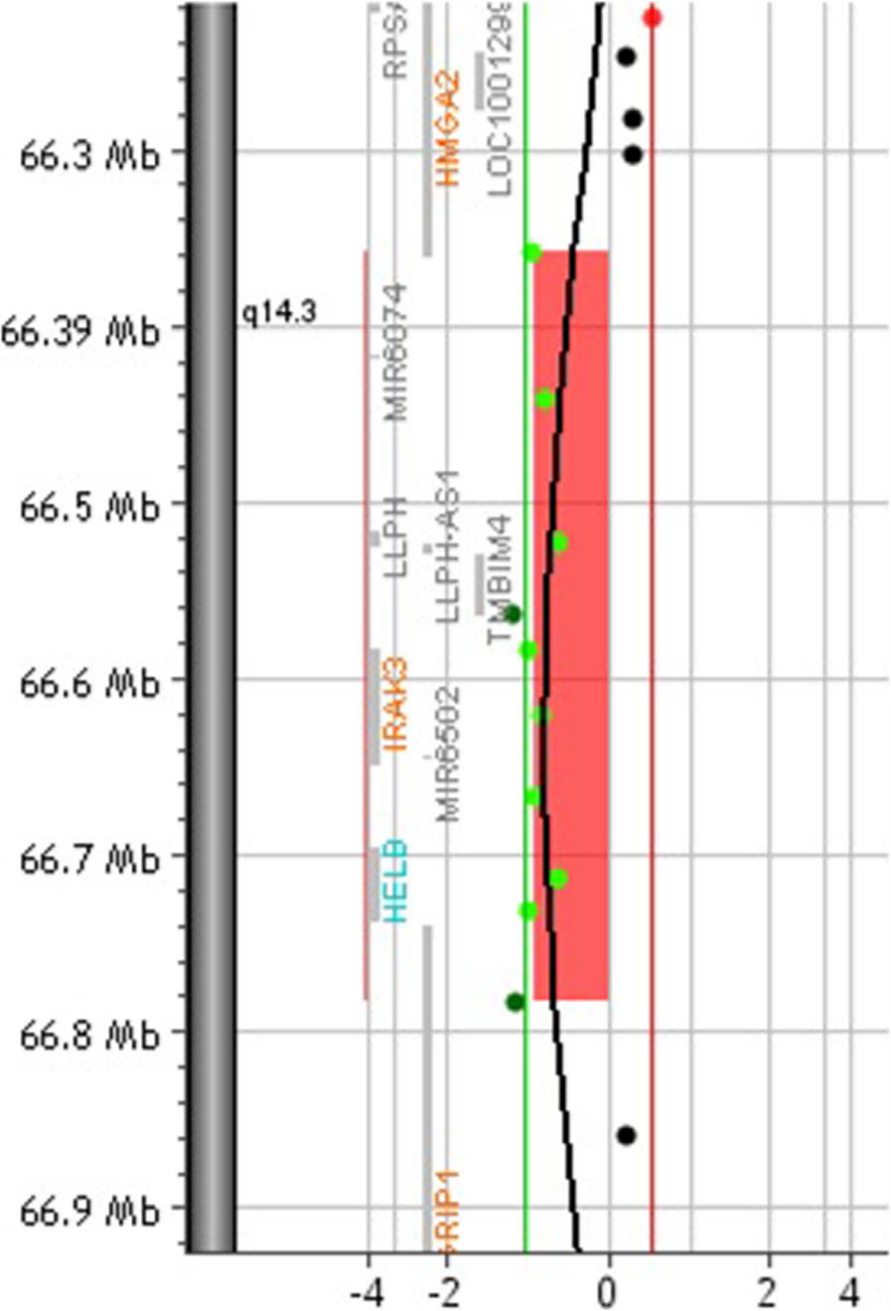
Genome-wide array-CGH analysis: 425 Kb deletion of the long arm of the chromosome 12, ranging from 66358287 Mb (12q14.3) to 66782791 Mb (12q14.3)

In accordance with the clinical suspicion of SRS assessed through the NHCSS, we proceeded according to the flow-chart proposed by Azzi et al. and performed the classical genetic test for this syndrome. The Methylation-Specific Multiplex Legation-dependent Probe Amplification (MS-MLPA) at the imprinting region 11p15 did not reveal Loss Of Metilation (LOM) and the study of microsatellite markers of chromosome 7 did not detect UniParental Disomy (UPD). Subsequently, consistently with the aforementioned flow-chart, we proceeded with the research of genome-wide microdeletions or microduplications by Array-CGH (Array-Comparative Genome Hybridization). This analysis showed a de novo partial deletion of the long arm of the chromosome 12, 425 Kb-sized, from position 66,358,287, in which there is the most terminal probe delete on the array (cytogenetic locus 12q14.3) to 66,782,791 (cytogenetic locus 12q14.3) (Fig. 2). According with the build UCSC Genome Browser (Hg build 19) several genes lie in this region including *the OMIM disease causing genes HMGA2 (OMIM 600698, partially involved), IRAK3 (OMIM 604459) and GRIP1 (OMIM 604597). This microdeletion is not reported in DECIPHER. However, the same database recorded a 1.67 Mb deletion* (ID 337828) of unknown significance, encompassing the same genes and maternally inherited, in a female infant with episodic hyperhidrosis, failure to thrive, moderate short stature, facial asymmetry, prominent forehead, proptosis, scoliosis and history of intrauterine growth retardation. A deletion with the same genomic and clinical features (size, genomic coordinates, maternal origin, clinical picture except for the absence of hyperhidrosis) have been previously described by Heldt et al. in a family where it segregated along with Silver-Russell like phenotype. Even if neither Heldt nor the database DECIPHER refer to the case reported by the other, we believe it is reasonably to conclude that the deletions reported in DECIPHER and in Heldt paper are the same [2, 13].

## Materials and methods

DNA of proband and his parents was extracted with QIAamp DNA blood Midi Kit (Qiagen Inc., Valencia, CA) according to the manufacturer’s instructions. The normal control was a commercially available male Promega (Promega Corporation, Madison, WI). Array-CGH analysis was performed using a Human Genome CGH Microarray Kit 8x60K (Agilent) with a resolution of 100–150 kb according to the manufacturer’s instructions. Array slides were analyzed with an Agilent G2505B scanner. Image were analyzed and visualized with CytoGenomics 3.0.6.6 (Agilent Technologies, Santa Clara, CA).

## Discussion & conclusions

More than 40% of patients with a SRS-like phenotype remain without an etiological diagnosis [3, 4]. Several genes have been proposed as candidate genes. During the last 10 years, different reports have suggested the role of *HMGA2* in the etiology of SRS-like phenotype. To our knowledge, only 28 cases with a SRS-like phenotype due to the haploinsufficiency of *HMGA2* have been reported (Table 1). In particular, 23 deletions, detected by a-CGH, have been described. The remaining 5 cases were: a 7 bp deletion [11], a nonsense variant and a frameshift variant [10] and 2 deletions of exon 2 [12] and exons 1–2 [8]. The 23 deletions described so far range between 387 kb and 10.12 Mb, and involve other contiguous genes [9, 12–23]. Despite the variable size of these deletions, a critical region of 2.61 Mb has been identified. The OMIM disease causing genes included in this region are: *LEMD3* (OMIM 607844), *MSRB3* (OMIM 613719), *HMGA2* (OMIM 600698), *IRAK3* (OMIM 604459), *GRIP1* (OMIM 604597).

**Table 1:** Summary of all cases with a SRS-like phenotype due to the haploinsufficiency of HMGA2 described in literature

	Sex	Genotype Microdeletion/Mutation	Inheritance	IUGR	SGA	Failure To Thrive	Relative macrocephaly	Asimmetry	Fifth-finger clinodactyly	Frontal bossing/prominent forehead	Triangular facies	Micrognathia/Narrow chin	Short stature	Language delay	Other developmental disorders	Osteopoikilosis
Menten et al. 2007 [9] - Case 1^a^	F	Del 6 Mb	Unknown	No	No	No	No	No	No	No	No	Yes	Yes	No	Delayd neuromotor development and learning difficulties	Yes
Menten et al. 2007 [9] - Case 2^a^	F	Del 6 Mb	Unknown	No	No	No	No	No	No	No	No	No	Yes	No	Learning difficulties and intellectual disability	Yes
Menten et al. 2007 [9] - Case 3^a^	M	Del 3,44 Mb	Unknown	Unknown	Yes	No	No	No	No	No	Yes	Yes	Yes	No	Delayd neuromotor development and learning difficulties	Yes
Mari et al. 2009 [14]	M	Del 1,83 Mb	De novo	Yes	Yes	No	No	No	No	Yes	Yes	Yes	Yes	Yes	Motor delay	No
Buysse et al. 2009 [12] - Case 1	M	Del exon 2	Maternal	No	No	Yes	No	No	No	No	No	No	Yes	No	No	No
Buysse et al. 2009 [12] - Case 2^a^	M	Del 8,95 Mb	De novo	Yes	Yes	Yes	No	No	No	Yes	No	No	Yes	No	Global developmental delay	No
Buysse et al. 2009 [12] - Case 3^a^	M	Del 3,48 Mb	De novo	Yes	Yes	Yes	No	No	No	No	No	No	Yes	No	Mild developmental delay	No
Spengler et al. 2010^a^ [15]	F	Del 1,35 Mb	De novo	Yes	Yes	Yes	Yes	No	Yes	Yes	Yes	No	Yes	Yes	No	No
Lynch et al. 2011 [16] -Case 1	F	Del 10,11 Mb	De novo	No	No	Yes	No	No	Yes	No	No	No	Yes	Yes	Developmental delay	No
Lynch et al. 2011 [16] - Case 2	F	Del 10,12 Mb	De novo	No	No	Yes	Yes	No	No	Yes	No	No	Yes	No	Intellectual disability, ASD	No
Bibb et al. 2012^a^ [17]	F	Del 3,2 Mb	Maternal	No	No	Yes	No	No	Yes	No	No	Yes	Yes	Yes	Mild intellectal disability and behavioural problems	No
Bibb et al. 2012 [17] - Mother^a^	F	Del 3,2 Mb	Unknown	Unknown	Yes	Yes	No	No	Yes	No	No	Yes	Yes	Yes	Learning disabilities	Yes
Alyaqoub et al. 2012^a^ [18]	F	Del 4,17 Mb	De novo	Yes	Yes	No	No	No	No	No	Yes	No	Yes	Yes	Hypotonia	No
Takenouchi et al. 2012 [19]	F	Del 4 Mb	De novo	Yes	Yes	Yes	No	No	No	No	No	No	Yes	No	No	No
Nso-Roca et al. 2014 [20]	F	Del 8,35 Mb	De novo	No	No	Yes	No	No	No	Yes	Yes	No	Yes	No	No	No
Mc Cormack et al. 2015^a^ [21]	M	Del 3,8 Mb	Unknown	Yes	No	No	Yes	No	No	No	No	No	Yes	No	ASD	No
Raymond et al. 2015 [22] (prenatal diagnosis)	M	Del 387 Kb t(1;12;14)(q42;q14;q32)	De novo	Yes	(ITG)	(ITG)	No	No	No	Yes	Yes	Yes	(ITG)	(ITG)	(ITG)	No
De Crescenzo et al. 2015 [11]	F	Del 7 bp at splicing site acceptor (intr 4)	Maternal	Unknow	Yes	Yes	Yes	No	Yes	Yes	No	Yes	Yes	Unknown	Unknown	No
Abi Habib 2018 [10] - Case 1	F	Nonsense mutation	De novo	Unknown	Yes	Yes	Yes	No	No	Yes	Yes	Yes	Yes	Unknown	Unknown	No
Abi Habib 2018 [10] - Case 2	M	Frameshift mutation	Unknown	Unknown	Yes	Yes	Yes	No	No	Yes	Yes	Yes	Yes	Unknown	Unknown	No
Fischetto et al. 2017^a^ [23] - Brother 1	M	Del 1,9 Mb	Maternal	Yes	Yes	No	No	No	No	No	Yes	No	Yes	No	No	Yes
Fischetto et al. 2017^a^ [23] - Brother 2	M	Del 1,9 Mb	Maternal	Yes	Yes	No	No	No	No	No	Yes	No	Yes	No	Developmental delay	No
Fischetto et al. 2017^a^ [23] - Mother	F	Del 1,9 Mb	Unkown	Yes	Yes	No	No	No	No	No	Yes	Yes	Yes	No	Motor delay	Yes
Leszinski et al. 2018 [8]	F	Del exon 1–2	De novo	Unknown	Yes	Yes	No	No	No	Yes	Yes	Yes	Yes	No	No	No
Heldt et al. 2018^a^ [13] - Sister 1	F	Del 1,67 Mb	Maternal	No	Yes	Yes	No	No	No	Yes	No	No	Yes	No	No	No
Heldt et al. 2018^a^ [13] - Brother	M	Del 1,67 Mb	Maternal	No	Yes	Yes	No	No	No	No	No	No	Yes	No	No	No
Heldt et al. 2018^a^ [13] - Mother	F	Del 1,67 Mb	Unknown	Unknown	Unknown	No	No	No	No	No	No	No	Yes	No	No	No
Our patient	M	Del 425 Kb	De novo	Unknown	Yes	Yes	Yes	Yes	Yes	Yes	Yes	Yes	Yes	Yes	No	No

^a^ Deletions involving also *LEMD3* gene

The *HMGA2* gene encodes for the HMGA2 protein, member of the HMGA family. This family of four proteins (HMGA1a, HMGA1b, HMGA1c and HMGA2), encoded by two genes (*HMGA1* and *HMGA2*), can interact with DNA altering its conformation and regulating the transcription of several genes. *HMGA2* regulates the transcription of the known fetal growth factor IGF2. Multiple studies on human and animal models have demonstrated a high expression of *HMGA2* during fetal period when it may play a crucial role in embryonic development and linear growth. Moreover, *HMGA2* has showed to be essential in regulating cell growth, proliferation, differentiation and death, in promoting adipocyte differentiation. Over-expression of *HMGA2* has also been found in some tumoral tissues (uterine leiomyoma, gastric cancer, pediatric lipoma) [6–8, 12, 24–27]. These experimental studies, added to the clinical data reported in literature, converge in proving the key role of *HMGA2* alterations in pre- and postnatal growth failure and failure to thrive that is otherwise unexplained.

As previously reported, *LEMD3* is the causative gene of Buschke-Ollendorff syndrome and Osteopoikilosis with or without melorheostosis (OMIM 166700). All patients with characteristic features of this syndrome result carrying deletions involving also *LEMD3*, as suggested by data in Table 1 (see superscripts and notes).

In regard to the other OMIM disease causing genes included in the critical region, biallelic mutations in *MSRB3* gene have been described in cases of deafness autosomal recessive 74 (OMIM 613718), while several polymorphisms of *IRAK3* have been associated with increased asthma susceptibility. The *GRIP1* gene, known as causative gene of Fraser syndrome transmitted in recessive manner, has been considered as candidate gene for neurodevelopmental disorders in most patients carrying 12q14.3 deletions. This hypothesis arises from the evidence of his high expression in brain tissue in both fetal and postnatal period, and his clear role in synaptic functioning. However, developmental disorders, such as language and/or motor delay, learning difficulties and intellectual disability, have also been described in patients with a 12q14.3 deletion not involving *GRIP1* gene [15, 23].

An exclusive role of *HMGA2* gene in the pathogenesis of SRS-like cases is supported by the evidence that pre- and postnatal growth failure and underweight, typical features of SRS (the first two items of the Netchine-Harbison CSS), have been described in all patients carrying different aberrations of *HMGA2* regardless of their dimension. In fact, there are not significant clinical difference among patients with more or less extensive deletions of the 12q14.3 region (involving multiple genes, as *HMGA2*), and patients with pathogenic variants or intragenic deletions of *HMGA2*. Moreover, recently a duplication involving *HMG2A* in a patient with overgrowth, obesity and tall stature with advanced bone age has been reported [28], confirming the role of *HMGA2* gene in growth regulation.

Regarding the other clinical features of the patients described in the literature (see Table 1), relative macrocephaly and a prominent forehead, two further items of the NHCSS, present in our patient, to our knowledge, were described in literature in less than half of the cases. Another item of the NHCSS, the body asymmetry, does not seem to be a typical feature of the 12q14.3 microdeletion syndrome, while it is reported in our proband, who has lower limbs asymmetry. To date, our case represents the only one 12q14.3 microdeletion syndrome with limb asymmetry. Finally, our patient presents a moderate speech delay, in the absence of other neurodevelopmental disorders. The presence of neurodevelopmental disorders (language and/or motor delay, learning difficulties, intellectual disability, behavioural problems), seems to be a distinct and frequent feature of 12q14.3 microdeletion syndrome, as it is reported in about half of patients (see Table 1) [2].

In conclusion, we suggest to investigate *HMGA2* gene in all the patients with evocative phenotype for SRS and negative genetic results for MS-MLPA at the imprinting region 11p15 and microsatellite markers of chromosome 7. In order to complete the diagnostic work-up of SRS we recommend array-CGH analysis, and subsequently the sequence analysis and the MLPA of *HMGA2* gene.

## Data Availability

The datasets used and/or analyzed during the current study are available from the corresponding author on reasonable request.

## Abbreviations

SRS: Silver-Russell syndrome
SD: Standard deviations
MRI: Magnetic resonance imaging
MS-MLPA: Metilation-Specific Multiplex Legation-dependent Probe Amplification
LOM: Lost Of Metilation
UPD: UniParental Disomy
a-CGH: Array-Comparative Genome Hybridization
